# Supplementary material for: G1 and G2 ApolipoproteinL1 modulate macrophage inflammation and lipid accumulation through the polyamine pathway
Source: bioRxiv. 2025 Jun 8:2025.06.06.658371. Preprint. [Version 1] doi: 10.1101/2025.06.06.658371 (PMC12157409; doi:10.1101/2025.06.06.658371)
Supplement: Supplement 1 [file NIHPP2025.06.06.658371v1-supplement-1.pdf]

bioRxiv preprint doi: https://doi.org/10.1101/2025.06.06.658371; this version posted June 9, 2025. The copyright holder for this preprint (which was not certified by peer review) is the author/funder, who has granted bioRxiv a license to display the preprint in perpetuity. It is made available under aCC-BY 4.0 International license.

Supplemental Figure 1

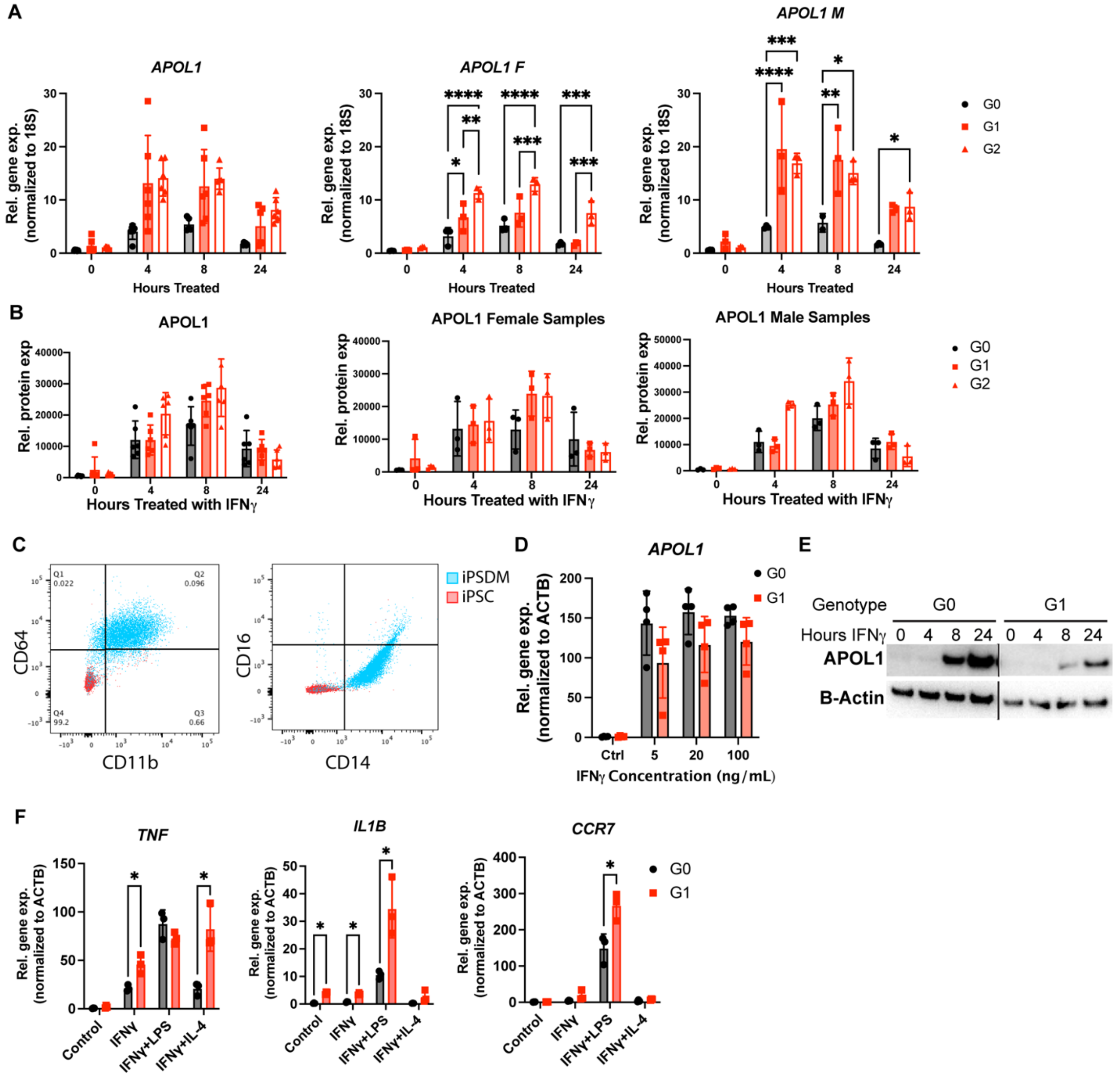

bioRxiv preprint doi: https://doi.org/10.1101/2025.06.06.658371; this version posted June 9, 2025. The copyright holder for this preprint (which was not certified by peer review) is the author/funder, who has granted bioRxiv a license to display the preprint in perpetuity. It is made available under aCC-BY 4.0 International license.

Supplemental Figure 1. APOL1 expression in BMDMs and iPSDM is increased with IFNγ

(A) Gene expression of *APOL1* measured with qRT-PCR in combined sex, female, and male BMDMs. **(B)** Quantification of APOL1 protein expressed measured with western blot in combined, female, and male BMDMs. **(C)** Flow cytometric analysis of CD11b, CD64, CD14, and CD16 surface expression on G1 iPSDM and G1 iPSCs. **(D)** Gene expression of *APOL1* measured in G0 and G1 iPSDMs treated with IFNγ. **(E)** western blot of APOL1 in G0 and G1 iPSDMs treated with IFNγ. BMDM experiments were performed in BMDMs from 3-6 mice per genotype per group, with both sexes represented. **(F)** Gene expression of *TNA, IL1B,* and *CCR7* measured with qRT-PCR in G0 and G1 iPSDMs treated with IFNγ (25 ng/mL), LPS (10 pg/mL), and IL-4 (10 ng/mL) for 24 hours. BMDM experiments in **(A, B)** were completed in BMDMs from 4 mice from each genotype and each sex. iPSDM experiments in **(C, D)** were performed in 2 separate differentiations across 2 independent experiments. Experiments in **(E)** were performed with 3 separate wells of iPSDM. Data are expressed as mean ± SD. *p<0.05, **p<0.01 ***p<0.005 ****p>0.001. 2-way ANOVA with Tukey's Multiple Comparison Test

bioRxiv preprint doi: https://doi.org/10.1101/2025.06.06.658371; this version posted June 9, 2025. The copyright holder for this preprint (which was not certified by peer review) is the author/funder, who has granted bioRxiv a license to display the preprint in perpetuity. It is made available under aCC-BY 4.0 International license.

Supplemental Figure 2

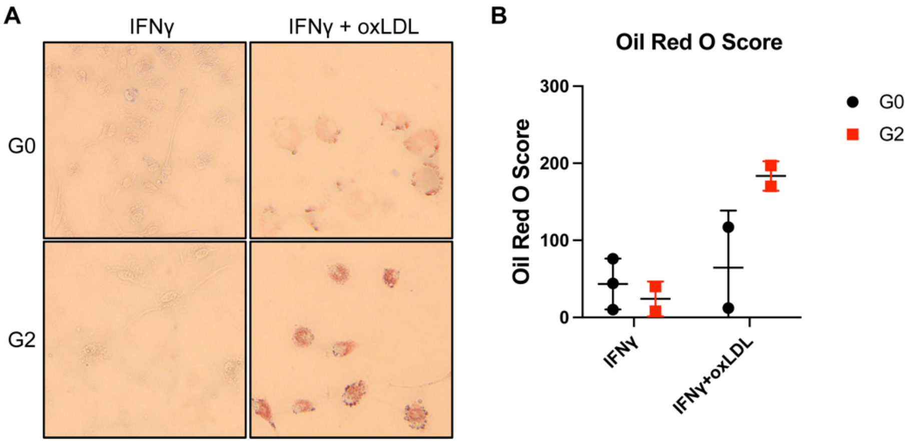

Supplemental Figure 2. G2 BMDMs from the C57Bl/6J background increase lipid accumulation with oxLDL incubation.

(A) Oil Red O (ORO) stained images of BMDMs in the presence or absence of 5ng/mL IFNγ and 50 µg/mL oxidized LDL (oxLDL) for 72 hours. BMDMs were generated from G0 and G2 mice from the C57Bl/6J background**. (B)** Quantification of the Oil Red O stain. Experiments were completed in BMDMs from 2-3 mice per genotype per group, with both sexes represented. Data are expressed as mean ± SD.

bioRxiv preprint doi: https://doi.org/10.1101/2025.06.06.658371; this version posted June 9, 2025. The copyright holder for this preprint (which was not certified by peer review) is the author/funder, who has granted bioRxiv a license to display the preprint in perpetuity. It is made available under aCC-BY 4.0 International license.

Supplemental Figure 3

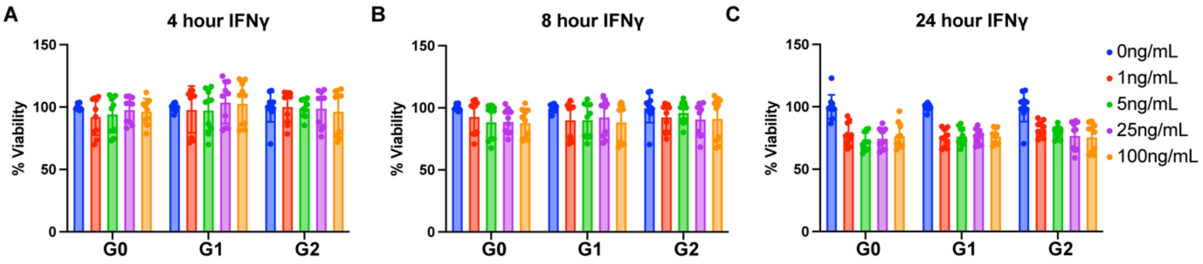

Supplemental Figure 3. Cell viability of BMDMs treated with IFNγ is slightly decreased at 24 hours

(A-C) Cell viability of BMDMs treated with 0-100 ng/mL IFNγ for 4, 8, and 24 hours measured with CellTiter-Glo. Experiments were performed using BMDMs from 8 mice across 2 independent experiments, with both sexes represented. Data are expressed as mean ± SD.

bioRxiv preprint doi: https://doi.org/10.1101/2025.06.06.658371; this version posted June 9, 2025. The copyright holder for this preprint (which was not certified by peer review) is the author/funder, who has granted bioRxiv a license to display the preprint in perpetuity. It is made available under aCC-BY 4.0 International license.

Supplemental Figure 4

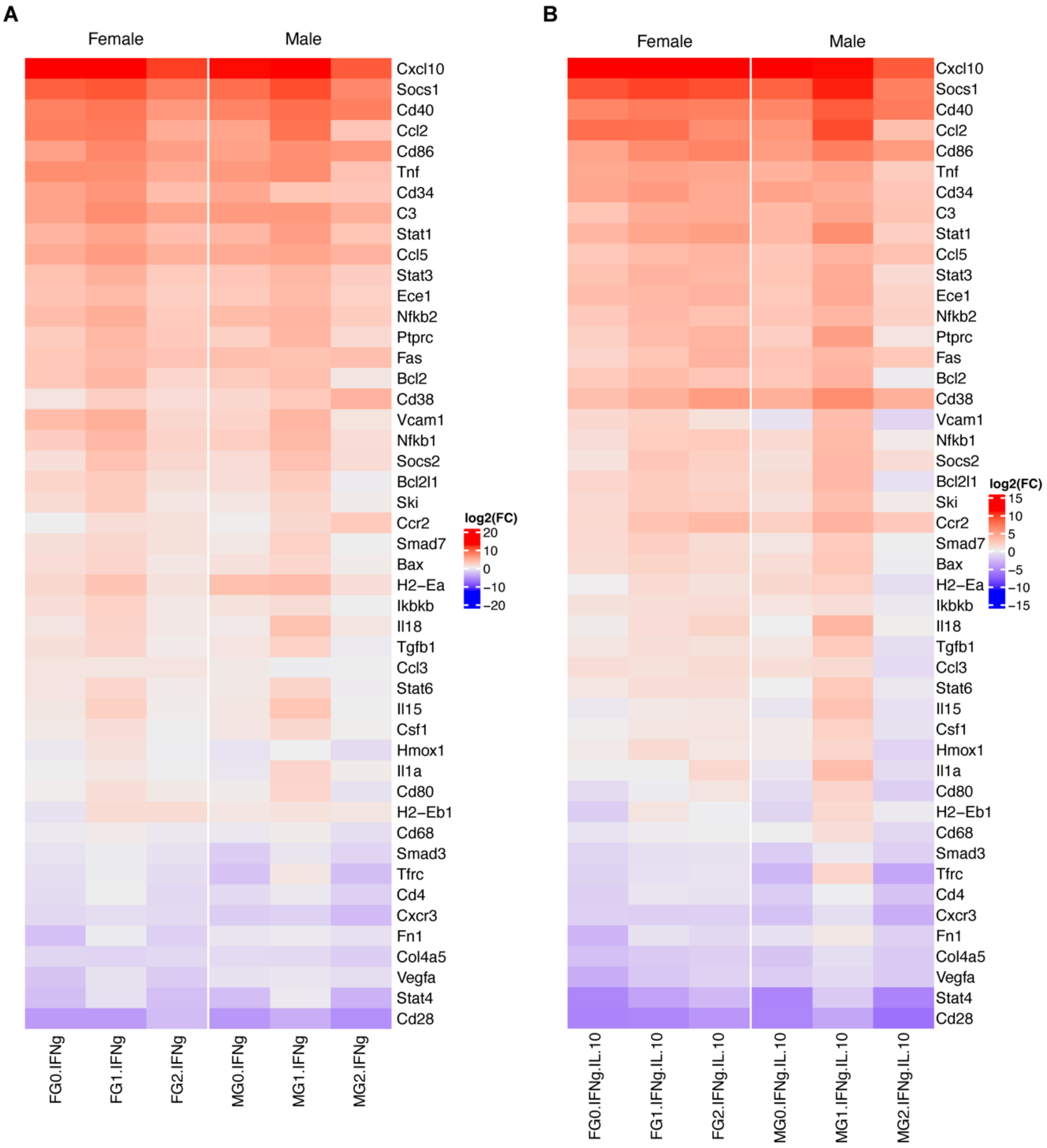

Supplemental Figure 4. qPCR array of immune genes in female and male BMDMs.

(A-B) qPCR array of immune-related genes in female and male BMDMs treated with IFNγ (5 ng/mL) and IL-10 (10 ng/mL). Experiment was completed in BMDMs from 2 mice per genotype per group.

bioRxiv preprint doi: https://doi.org/10.1101/2025.06.06.658371; this version posted June 9, 2025. The copyright holder for this preprint (which was not certified by peer review) is the author/funder, who has granted bioRxiv a license to display the preprint in perpetuity. It is made available under aCC-BY 4.0 International license.

Supplemental Figure 5

A

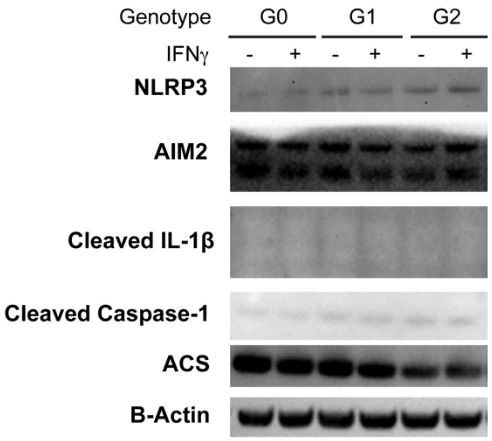

Supplemental Figure 5. NLRP3 inflammasome proteins are not activated with IFNγ treatment in APOL1 BMDMs.

(A) Representative western blot of NLRP3, AIM2, cleaved IL-1β, cleaved Caspase-1, and ACS in G0, G1 and G2 BMDMs treated with IFNγ (5 ng/mL) or control. Experiments were performed in BMDMs from 4 mice per genotype per group, with both sexes represented.

bioRxiv preprint doi: https://doi.org/10.1101/2025.06.06.658371; this version posted June 9, 2025. The copyright holder for this preprint (which was not certified by peer review) is the author/funder, who has granted bioRxiv a license to display the preprint in perpetuity. It is made available under aCC-BY 4.0 International license.

Supplemental Figure 6

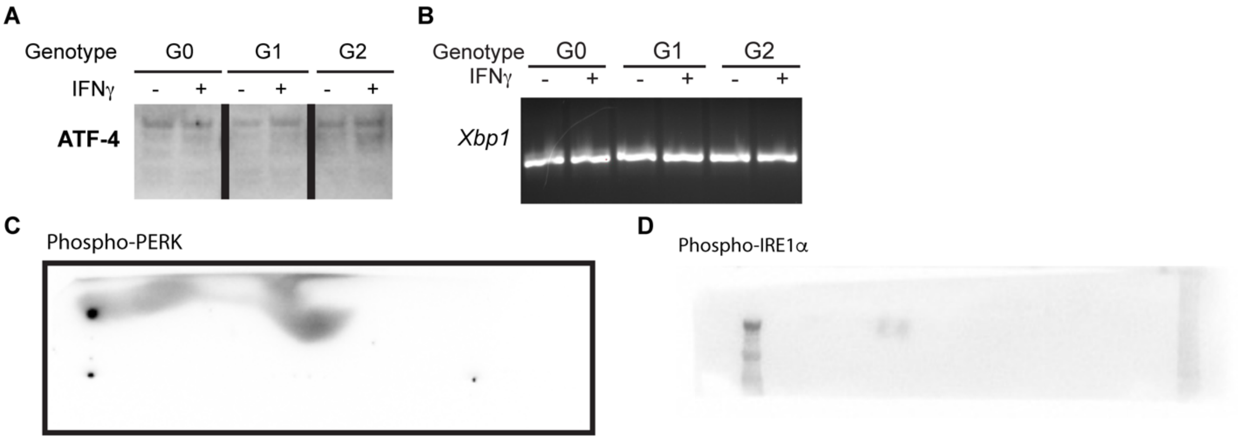

Supplemental Figure 6. ER stress proteins and genes are not expressed in APOL1 BMDMs with IFNγ treatment.

Representative western blot of ATF4 **(A)**, phospho-PERK **(C)**, phospho-IRE1α **(D)** and RT-PCR of Spliced *Xbp1* **(B)** in G0, G1 and G2 BMDMs treated with IFNγ (5 ng/mL). Experiments were completed in BMDMs from 4 mice per genotype per group, with both sexes represented.

bioRxiv preprint doi: https://doi.org/10.1101/2025.06.06.658371; this version posted June 9, 2025. The copyright holder for this preprint (which was not certified by peer review) is the author/funder, who has granted bioRxiv a license to display the preprint in perpetuity. It is made available under aCC-BY 4.0 International license.

Supplemental Figure 7

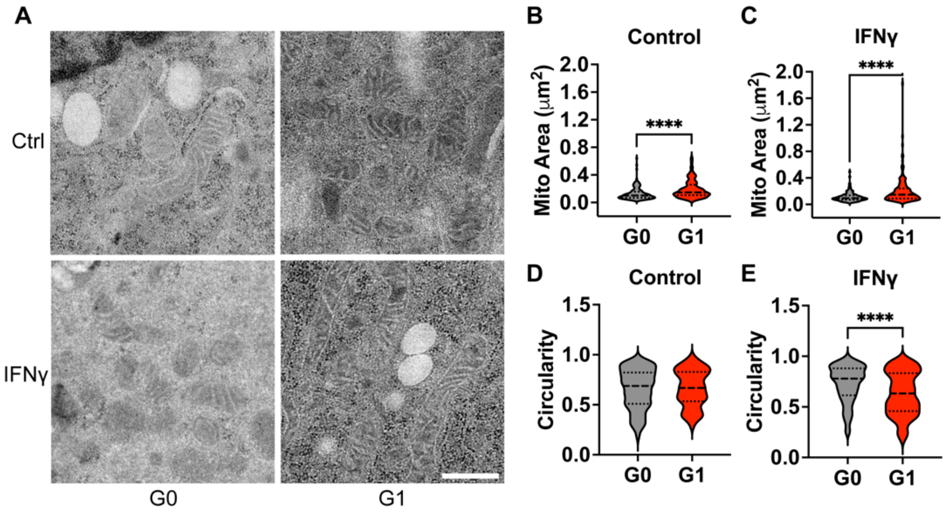

Supplemental Figure 7. Mitochondrial area is increased in G1 iPSDM compared to G0

G0 and G1 iPSDMs were treated with 20 ng/mL IFNγ for 8 hours.TEM images were collected of at least 20 cells and over 100 mitochondria were quantified for area and circularity. **(A)** Representative images of mitochondria in control and treated iPSDM. **(B, C)** Quantification of mitochondrial area in iPSDM. **(D, E)** Quantification of mitochondrial circularity in iPSDM. Experiments were performed in iPSDM from 4 separate differentiations across 2 independent experiments. Data are expressed as median and interquartile range. Unpaired *t* test. ****p <0.001

bioRxiv preprint doi: https://doi.org/10.1101/2025.06.06.658371; this version posted June 9, 2025. The copyright holder for this preprint (which was not certified by peer review) is the author/funder, who has granted bioRxiv a license to display the preprint in perpetuity. It is made available under aCC-BY 4.0 International license.

Supplemental Figure 8

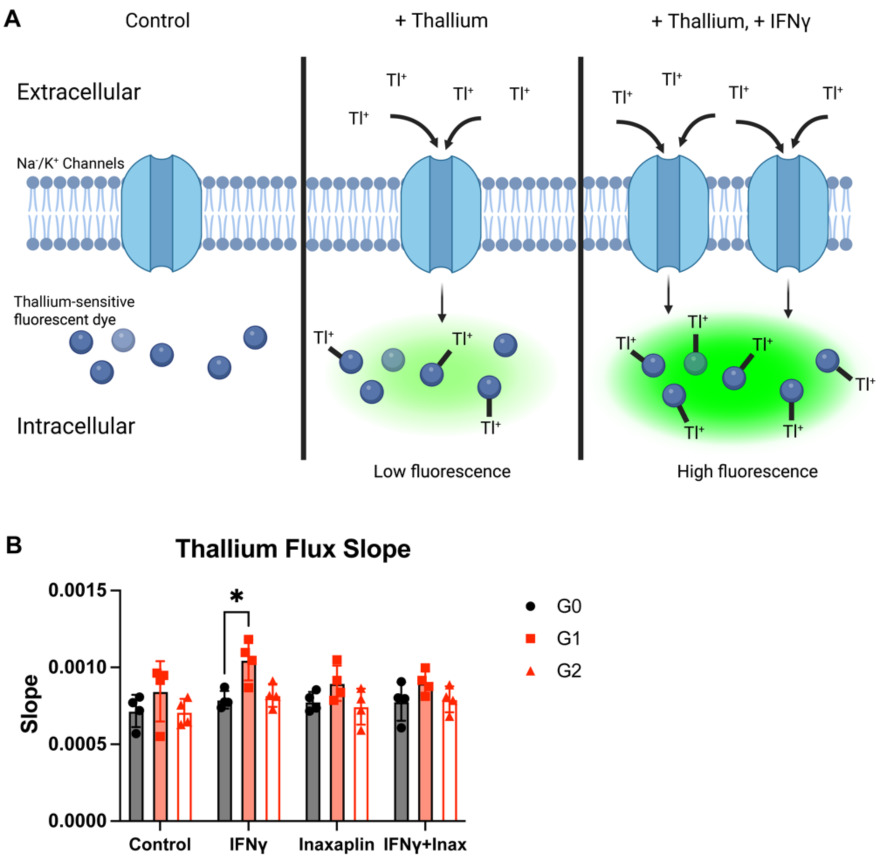

Supplemental Figure 8. Thallium flux in APOL1 BMDMs is unchanged with IFNγ and VX-147 treatment.

(A) Diagram of Thallium flux assay. **(B)** Slope of fluorescence after thallium addition to G0, G1 and G2 BMDMs treated with IFNγ (5 ng/mL), VX-147(1 μM) or DMSO control. Experiments were performed in BMDMs from 4 mice per genotype per group, with both sexes represented. Data are expressed as mean ± SD. Unpaired *t* test. *p <0.05
